# Identification of novel Tet(X6)-Tet(X2) recombinant variant in *Elizabethkingia meningoseptica* from a bullfrog farm and downstream river in China

**DOI:** 10.3389/fmicb.2024.1453801

**Published:** 2024-07-31

**Authors:** Haobo Jin, Qing Jia, Xi Jin, Xinlong Zhu, Min-Ge Wang, Ruan-Yang Sun, Chaoyue Cui

**Affiliations:** ^1^Laboratory Animal Centre, Wenzhou Medical University, Zhejiang, China; ^2^Department of Clinical Laboratory, The Second Affiliated Hospital of Wenzhou Medical University, Zhejiang, China; ^3^Phage Research Center, Liaocheng University, Liaocheng, Shandong, China; ^4^National Risk Assessment Laboratory for Antimicrobial Resistance of Animal Original Bacteria, South China Agricultural University, Guangzhou, Guangdong, China

**Keywords:** *tet*(X), recombinant variant, *Elizabethkingia meningoseptica*, ICE, tigecycline

## Abstract

**Introduction:**

The dissemination of strains producing tetracyclines monooxygenase Tet(X) from breeding farms to the natural environment poses a potential threat to public health.

**Methods:**

Antimicrobial susceptibility testing and WGS were performed to identify resistance phenotypes and genotypes. Cloning experiments, sequence alignment, and homology modeling were used to characterize the function and formation mechanisms of the recombinant variant. The mobilization potential of Tet(X) was assessed by collinearity analysis, conjugation experiments, and phylogenetic analysis.

**Results:**

Three *tet*(X)-producing *Elizabethkingia meningoseptica* strains were isolated from bullfrog breeding ponds, the sewage outlet, and downstream river in Zhejiang Province, China. These strains carry a novel Tet(X) variant, differing from Tet(X6) by seven residues, and possess the ability to degrade tetracyclines. Interestingly, the novel Tet(X) is a recombinant variant formed by homologous recombination of Tet(X6) and the C-terminal of Tet(X2). Further analysis revealed that Tet(X6) formed several Tet(X) variants, including Tet(X5), through homologous recombination. The novel *tet*(X) gene is located on a circularizable integrative and conjugative element (ICE*EmeChn3*), with IS*wz1* participating in the recombination of its multi-drug resistance region, potentially facilitating the mobilization and recombination of *tet*(X) in early hosts. These three strains were clonally transmitted and shared a close genetic relationship (SNP < 62) with a clinically-sourced strain isolated from the same province.

**Discussion:**

To our knowledge, this is the first report of homologous recombination between Tet(X) variants with differing activities. These clonal strains provide evidence of the transmission of *tet*(X)-positive strains from aquaculture sewage to the natural environment, highlighting the need to strengthen the monitoring and management of this emerging farming model.

## Introduction

*Elizabethkingia meningoseptica* is a non-motile, non-fermentative, oxidase-positive, Gram-negative bacterium that is widely present in both natural and hospital environments (Moore et al., 2016; Lin et al., 2019; Hu et al., 2023). As an emerging nosocomial pathogen, the detection rates of *E. meningoseptica* have been increasing in clinical settings in recent years. It can cause severe infectious diseases such as meningitis, sepsis, and lung infections, in immunocompromised patients, often resulting in high mortality rate ranging from 11 to 66% (Hu et al., 2023; Ma et al., 2023). *E. meningoseptica* is also considered a zoonotic pathogen as it has been found to infect multiple aquatic species such as fish, bullfrogs (Tsai et al., 2023), and turtles (Li et al., 2021). Furthermore, *E. meningoseptica* has intrinsic resistance to various antibiotics, including β-lactams, aminoglycosides, fluoroquinolones, and even peptide and carbapenems, which poses challenges in clinical treatment and increases the risk of patient mortality. Studies have shown that trimethoprim-sulfamethoxazole and minocycline, a member of the tetracyclines, are the few options for treating *E. meningoseptica* infections (Chan et al., 2019; Ma et al., 2023).

Tet(X) encodes a flavin-dependent monooxygenase (FMO) that confers resistance to all tetracyclines, including tigecycline, and leads to reduced sensitivity to the new-generation eravacycline and omamacycline (He et al., 2019; Hu et al., 2019; Cui et al., 2022a). Since the report of novel plasmid-mediated high-level *tet*(X) variants in 2019 (He et al., 2019; Sun et al., 2019), *tet*(X)-positive strains have been reported in 23 countries on six continents (Zhang S. et al., 2022). The *tet*(X) genes have been detected in various pathogenic bacteria, including *Klebsiella pneumoniae* (Liu et al., 2022), *Salmonella enterica* (Zhang Z. et al., 2022), *Pseudomonas aeruginosa* (Gasparrini et al., 2020), and *Proteus mirabilis* (Liu et al., 2020), among others, exhibiting significant sequence polymorphisms and a rapid spread trend. IncX1, IncQ, IncFII, and related hybrid plasmids are dominant plasmids carrying *tet*(X) and play a crucial role in facilitating the horizontal transfer and subsequent dissemination of *tet*(X) across strains, species and various potential ecological niches (Hu et al., 2019; Li et al., 2020; Cui et al., 2022b). Concerningly, there has been an increasing number of strains found to co-carry both *tet*(X) and carbapenemases or colistin resistance gene, *mcr*, sometimes even on the same plasmid (Cui et al., 2020; Mohsin et al., 2021; Xu et al., 2021; Sun L. et al., 2023; Li et al., 2024).

In recent years, bullfrog, an aquaculture food animal, has gained increasing popularity among young consumers due to its tender meat, delicious taste, and rich nutrition (Sun Q. et al., 2023). With the continuous rise in market demand, the scale of bullfrog breeding in China has steadily increasing year by year. Reports indicate significant growth in the total production of bullfrogs in China, from approximately 150,000 tons in 2013 to around 600,000 tons in 2021 (Lin et al., 2023). However, there is limited knowledge about the farming practices, presence of pathogens, and antibiotic resistance genes (ARGs), and their distribution characteristics in Chinese bullfrog farms, highlighting the need for improved supervision and monitoring in this area. This study focuses on typical bullfrog farms in Zhejiang Province, China, aiming to assess the dissemination characteristics of tigecycline-resistant strains along the sewage flow direction during bullfrog farming. To our knowledge, this is the first report of a novel *tet*(X) variant in *E. meningoseptica*, further limiting the clinical treatment options for infections caused by this species. The results of this study provide valuable insights into the evolutionary dynamics of *tet*(X) variants and highlight the need for comprehensive efforts to reduce the dissemination of resistance genes from bullfrog farms to the natural environment.

## Materials and methods

### Sample collection and identification of tet(X)-positive strains

In November 2023, we collected samples from three typical bullfrog farms in Wenzhou city, Zhejiang Province, China. These farms are situated on different tributaries of a large river, each approximately 5–10 km apart. Sampling was conducted with prior permission from the farm owners. In total, 36 samples were collected from each frog farm, comprising bullfrog fecal samples (*n* = 20), soil samples (*n* = 3), and water samples (*n* = 13). The 13 water samples were collected from various sources, including the inlet (*n* = 3), breeding ponds (*n* = 3), sewage channels (*n* = 3), sedimentation tank (*n* = 1), sewage outlet (*n* = 1), and downstream rivers (*n* = 2). Thus, a total of 108 samples were collected from the three bullfrog farms. A portion of samples was transferred into LB broth for bacterial enrichment, followed by inoculation onto LB agar plates supplemented with 2 mg/L tigecycline using inoculation loops. The strains were identified using 16 s DNA PCR and Sanger sequencing.

### Antimicrobial susceptibility testing

Antimicrobial susceptibility testing was conducted using the agar dilution method to determine the minimum inhibitory concentrations (MICs) for the isolates against the following antibiotics: amikacin (AMK), gentamicin (GEN), fosfomycin (FOS), rifampicin (RIF), tetracycline (TET), ampicillin (AMP), cefoxitin (FOX), cefotaxime (CTX), meropenem (MEM), trimethoprim-sulfamethoxazole (SXT), and florfenicol (FFC). The results were interpreted according to the guidelines of the Clinical and Laboratory Standards Institute (CLSI, 2020). Fresh Mueller-Hinton (MH) broth was prepared, and the MICs of tigecycline (TGC) and colistin (CS) were examined by the broth microdilution method. The breakpoints for tigecycline and colistin were interpreted according to the European Committee on Antimicrobial Susceptibility Testing (EUCAST, 2024) (resistant, >0.5 mg/L and > 2 mg/L), respectively. *E. coli* ATCC 25922 served as the quality control strain.

### Conjugation experiments

To verify the transferability of ICE*EmeChn3*, where *tet*(X) is located, rifampicin-resistant *Chryseobacterium* strains 1–1 and 2–2 from clinical sources were used as recipient strains and examined with the filter mating method. The donor and recipient strains were combined in a ratio of 1:3 and deposited onto a filter with a sterilized pore size of 0.22 μm. Subsequently, the mixture was cultured at 30°C for 24 h. Transconjugants were selected using LB agar supplemented with tigecycline (4 mg/L) and rifampin (300 mg/L). If potential transconjugants emerged, confirmation was conducted through PCR and Sanger sequencing.

### Multiple-sequence alignments and structure modeling

The novel Tet(X) was used as a query to perform a protein BLAST search with default options. Excluding model proteins, a total of 97 unique Tet(X)-like sequences were obtained with cutoff values of 75% sequence identity and 90% query coverage. In addition to these 97 retrieved sequences, 9 Tet(X) sequences were manually added. Multiple-sequence alignment was performed using MAFFT (v7.0) (Rozewicki et al., 2019) with default parameters. Subsequently, 320 conserved amino acid sites were used for phylogeny reconstruction using the Neighbor-Joining method. The JTT model was selected, and 100 bootstrap resampling were performed. The tree was visualized using Figtree (v1.4.4). The ESPript 3.0 was utilized to generate amino acid sequence alignments for Tet(X) variants (Robert and Gouet, 2014). Using the Tet(X7) crystal structure (PDB: 6WG9) as a template, the model structures of the novel Tet(X) were obtained through Modeller v9.24.

### Cloning and expression of Tet(X) protein

The novel *tet*(X) variant was cloned into the pBAD24 expression system to verify its functionality as previously reported, with minor changes. In brief, the pBAD24 expression vector was linearized through PCR amplification. Subsequently, PCR was used to amplify the full-length sequence of the novel Tet(X), adding 20 bp homologous arms identical to the linearized vector at both ends. Using the homologous recombination kit (Vazyme, China), ligate the vector and target gene fragments following the instruction manual. The constructed ligation product was transferred into competent *E. coli* Top10 cells using a chemical transformation method. Transformants were screened on LB plates containing ampicillin at a concentration of 100 mg/L. The transformants were subjected to tetracyclines MICs determination using the method mentioned above in 2.2.

### Whole-genome sequencing

The genomic DNA of the three *tet*(X)-positive *E. meningoseptica* strains was extracted using the TIANamp Bacteria DNA kit (Tiangen, China). Subsequently, sequencing was performed on an Illumina HiSeq 2500 platform (Bionova Biotech Co., Illumina). The obtained sequences were assembled using SPAdes version 3.12.0. Antibiotic resistance gene prediction was carried out using ResFinder version 3.1,^1^ and transposon and insertion (IS) element mining were conducted using ISfinder.^2^ ICEberg 2.0 is used to predict integrative and conjugative elements (ICEs) in bacterial genome sequences (Liu et al., 2019). Functional annotation was performed using the NCBI Prokaryotic Genome Annotation Pipeline server and RAST server. Strain CW3-1 was selected for long Nanopore system length sequencing, followed by assembly using Unicycler 0.4.1. Easyfig was used for IC*EmeChn3* collinearity analysis.

### Phylogenomic analysis

We downloaded the complete genome sequences of all 79 *E. meningoseptica* strains from the NCBI genome database and conducted phylogenetic analysis to assess their relatedness with the three *E. meningoseptica* strains isolated in this study. CSI phylogeny was used to generate concatenated alignments of high-quality single nucleotide polymorphisms (SNP) based on standard settings (Kaas et al., 2014) using the strain G4120 (GCF_002022145.1) as a reference sequence. The phylogenetic analysis was performed using PhyML v3.0^3^ to generate a maximum likelihood phylogenetic tree. The tree was visualized and decorated using iTOL V4 (Letunic and Bork, 2019).

## Results

### Identification of tet(X)-positive *Elizabethkingia meningoseptica* strains

In November 2023, three tigecycline-resistant strains (CW3-1, PW1, and RW1-1) were isolated from breeding ponds (CW), sewage outlets (PW), and downstream river (RW) at a bullfrog farm in Zhejiang Province, China. Subsequently, the presence of *tet*(X) in the three strains was confirmed by PCR testing. These three strains exhibited consistent morphologies on LB agar, displaying smooth surfaces with well-defined, plump, and neat orange-colored colonies. Further identification through 16S DNA and Sanger sequencing confirmed their classification as *E. meningoseptica* species. Antibiotic susceptibility testing revealed consistent resistant phenotypes among CW3-1, PW1, and RW1-1 strains. They exhibited high-level resistance to clinical drugs such as amikacin, gentamicin, fosfomycin, tetracycline, ampicillin, cefoxitin, cefotaxime, trimethoprim-sulfamethoxazole, and florfenicol, with susceptibility only to rifampicin (Table 1). Notably, CW3-1, PW1, and RW1-1 also exhibited resistance to tigecycline, meropenem, and colistin, three critical last-line drugs. Moreover, these *E. meningoseptica* strains also exhibited reduced sensitivity to the new-generation glycylcycline, eravacycline, thereby potentially leading to a scenario of no available drugs in clinical infections.

**Table 1:** MICs (mg/L) of 14 antimicrobials for 3 *tet*(X)-producing *Elizabethkingia meningoseptica* isolates.

	GEN	AMK	S/T	FFC	RIF	FOS	AMP	CTX	FOX	TET	MEM	CS	TGC	ERA
CW3-1	64	64	320	64	8	>256	256	64	64	128	16	>256	8	2
PW1	64	64	320	64	8	>256	256	64	64	128	16	>256	8	2
RW1-1	64	64	320	64	8	>256	256	64	64	128	16	>256	8	2
25,922	2	2	5	2	4	2	4	0.125	0.5	2	0.03	0.25	0.06	0.03

GEN, gentamicin; AMK, amikacin; S/T, trimethoprim/sulfamethoxazole; FFC, florfenicol; RIF, rifampicin; FOS, fosfomycin; AMP, ampicillin; CTX, cefotaxime; FOX, cefoxitin; TET, tetracycline; MEM, meropenem; CS, colistin; TGC, tigecycline; ERA, eravacycline.

### Characterization of novel tet(X) recombinant variant

**Figure 1:**
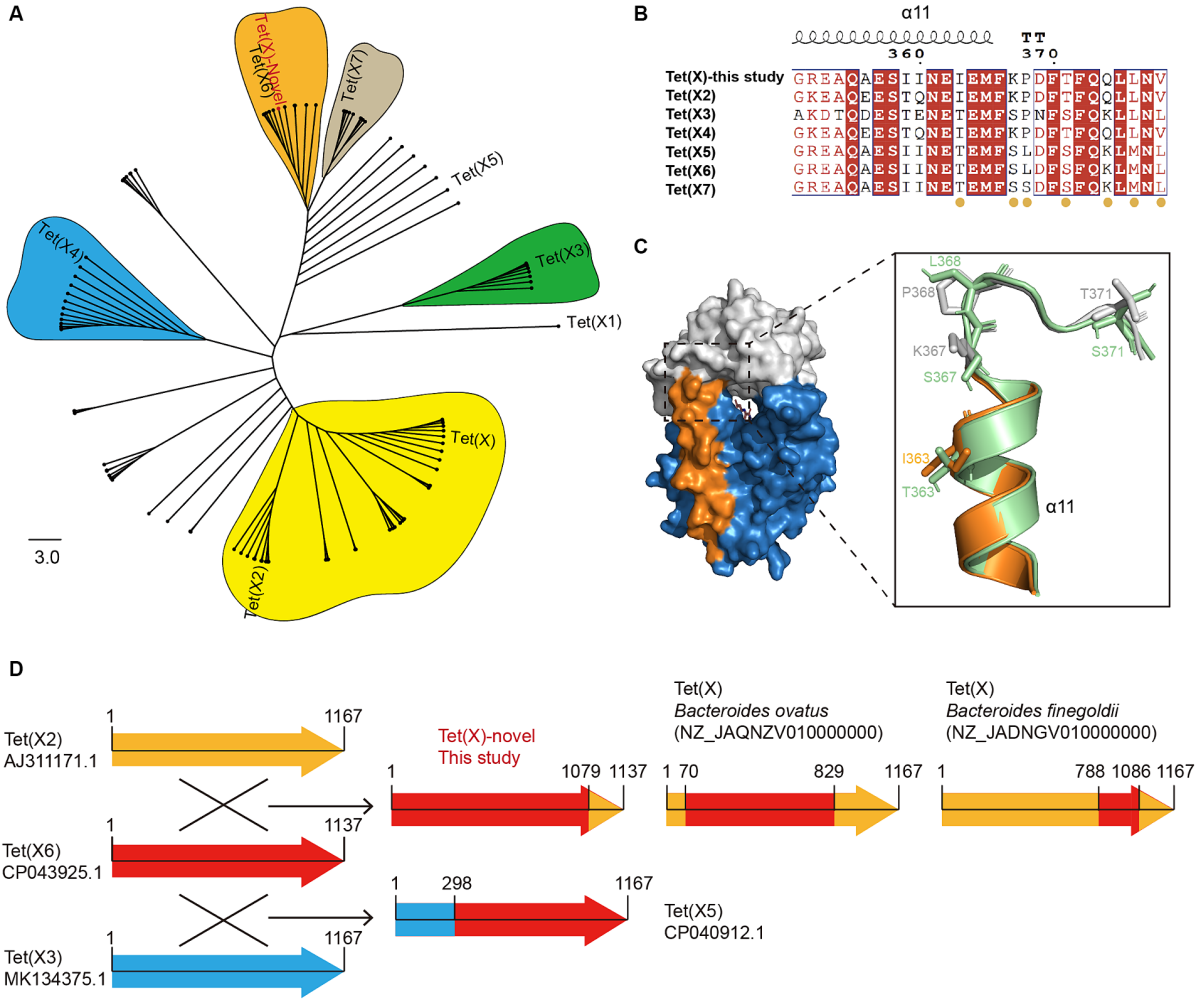
Characterization of novel tet(X) hybrid variant. **(A)** A Neighbor-Joining phylogenetic tree analysis was performed on 106 homologous Tet(X)sequences, with tet(X1) serving as the root of the tree. The clusters containing Tet(X/X2), Tet(X3), Tet(X4), Tet(X6), and Tet(X7) are represented by the colors yellow, green, blue, orange, and gray, respectively. **(B)** Alignment of C-terminal amino acid sequence of the novel Tet(X) hybrid variant with Tet(X2), Tet(X3), Tet(X4), Tet(X5), Tet(X6), and Tet(X7). The seven differential residues are marked below with orange circles. **(C)** The surface structure on the left is the homology model of the novel Tet(X) variant. The cartoon structure on the right is the C-terminal alignment of the novel Tet(X) with Tet(X7). The tetracycline binding region, FAD binding region and C-terminal α-helix of novel Tet(X) variant are colored by gray, blue, and orange, respectively. Tet(X7) is shown in cyan. **(D)** The illustration depicts homologous recombination among Tet(X) variants. Tet(X2), Tet(X6), and Tet(X3) are represented by the colors orange, red, and blue arrows, respectively.

Illumina short-read sequencing revealed that the three *E. meningoseptica* strains carried a novel Tet(X) variant, with a length of 1,137 bp, encoding a putative monooxygenase consisting of 378 amino acids. The novel Tet(X) variant exhibited amino acid similarities of 86.54, 78.89, 89.18, 93.14, 98.15, and 93.14% to previously reported major variants Tet(X2) (AJ311171.1), Tet(X3) (MK134375.1), Tet(X4) (MK134376.1), Tet(X5) (CP040912.1), Tet(X6) (CP043925.1), and Tet(X7) (CP025402.1), respectively. The phylogenetic tree reveals a clear divergence trend among Tet(X) variants (Figure 1A), among them, Tet(X6) represents an evolutionarily active variant with extensive genetic polymorphism distributed across multiple species. Tet(X)-novel shares the closest evolutionary relationship with Tet(X6), differing by seven amino acid residues, and both are clustered within the same evolutionary cluster.

Furthermore, the NCBI blastn search shows that the novel Tet(X) has 100% amino acid similarity with Tet(X) carried by a *Proteus mirabilis* (WP_159262696.1) strain isolated in 2020. To validate the functionality of the novel Tet(X), we cloned it into the pBAD24 vector and transformed it into *E. coli* TOP10 cells to characterize its ability to degrade tetracyclines. The MICs of tetracycline, doxycycline, minocycline, tigecycline, and eravacycline for *E. coli* Top10-pBAD24-Tet(X)-novel were determined to be 32, 16, 4, 4, and 2 mg/L, respectively (Supplementary Table S1). Compared to the control strain, the MIC values were increased by 16–250 folds, indicating its tetracyclines-degrading ability. Overall, the activity of the novel Tet(X) is comparable to that of Tet(X6), slightly lower than Tet(X3) and Tet(X4), and higher than the earlier variants Tet(X/X2). Given the unavailability of crystallographic data for Tet(X6) in the PDB database, we conducted homology modeling of the novel Tet(X) utilizing the crystal structure of Tet(X7) as a template, owing to its high similarity. The structural analysis indicates that all seven differing residues between the novel Tet(X) and Tet(X6) are all located at the C-terminus of the protein structure (Figure 1B). Among them, residue I363 is located on the 11th α-helix of Tet(X), far away from the substrate and flavin adenine dinucleotide (FAD) binding region (Figure 1C). Previous research has established a correlation between tetracyclines hydrolytic activity of Tet(X) and specific mutations occurring at residues 339, 340, 350, and 351 on the C-terminal α-helix (Cui et al., 2021). The remaining six differing residues are K367, P368, T371, Q374, L376 and V378, and all located on the C-terminal loop of Tet(X) (Figure 1C). However, due to the uniqueness of protein crystal structures, the crystallographic data for both N-terminal and C-terminal loops are often missing, rendering these regions unanalyzable.

### Identification of homologous recombination between Tet(X) variants

Because all seven differing residues between the novel Tet(X) and Tet(X6) are located at the C-terminal of the protein, and they match perfectly with corresponding residues in Tet(X2) and Tet(X4), there is a reasonable suspicion that the novel Tet(X) may have formed through homologous recombination between Tet(X6) and the C-terminal of Tet(X2/X4). The alignment results of the *tet*(X) genes’ nucleotide sequences provide evidence to support this hypothesis. It was observed that nucleotide positions 1–1,079 and 1,080–1,137 of the novel Tet(X) showed 100% nucleotide similarity to positions 1–1,079 of Tet(X6) and positions 1,110–1,167 of Tet(X2) respectively (Figure 1D). Significantly, upon further comparison to the Tet(X) variants documented in the NCBI database, an intriguing discovery emerges: homologous recombination events between these variants are more prevalent than previously believed. For example, the nucleotide positions 1–70 and 829–1,167 of the Tet(X) carried by the *B. ovatus* strain 2225st1 (NZ_JAQNZV010000000) exhibit a complete match with Tet(X2), while positions 71–828 show a complete match with Tet(X6). Furthermore, in the Tet(X) variant identified in *B. finegoldii* strain BSD2780120874b (NZ_JADNGV010000060), nucleotide positions 1–788 and 1,086–1,167 display complete alignment with Tet(X2), while positions 789–1,085 correspond to Tet(X6) with only four nucleotide differences, which do not result in amino acid changes. Moreover, the Tet(X5) variant, previously reported in *A. baumannii* (Wang et al., 2019), is a product of recombination between Tet(X3) and Tet(X6). Specifically, nucleotide positions 1–298 and 299–1,167 of Tet(X5) perfectly match the corresponding nucleotide sequences of Tet(X3) and Tet(X6) respectively (Figure 1D). These findings indicate the widespread involvement of Tet(X6) in homologous recombination among Tet(X) variants. As far as we know, this is the first observation of homologous recombination events occurring between different Tet(X) variants.

### Characteristics of tet(X)-harboring ICEEmeChn3

**Figure 2:**
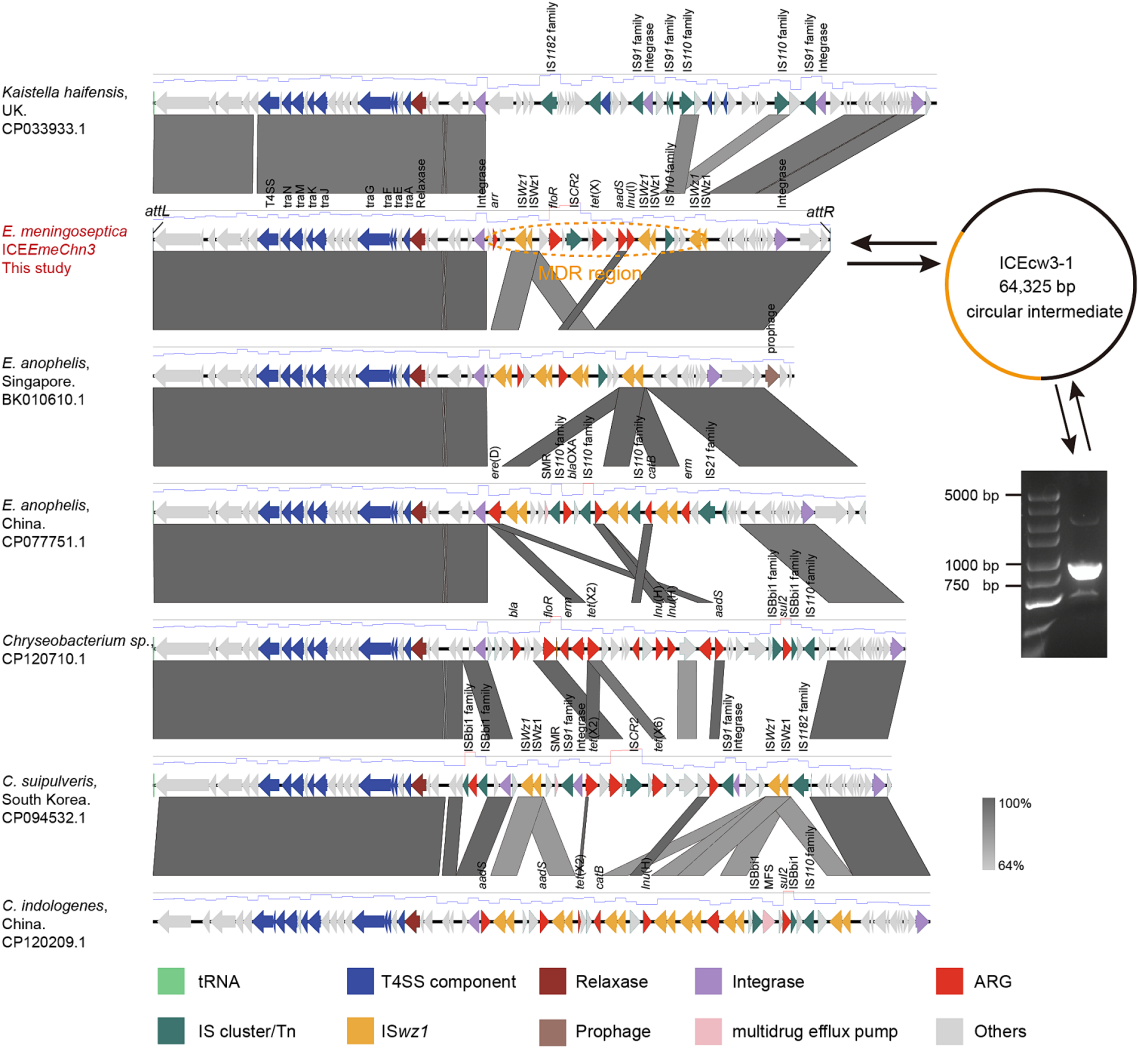
Linear sequence comparison of *tet*(X)-harboring ICE*EmeChn3*. Regions of homology are marked by shading. Arrows show the direction of transcription of open reading frames. T4SS component, antimicrobial resistance, transposases, etc., are marked according to the colors in the legend to indicate their functions.

To acquire the comprehensive genetic profile of *tet*(X), long-read Nanopore sequencing was conducted on CW3-1. The results indicated that the CW3-1 strain lacked any plasmids and had a single chromosome with a total length of 4,057,321 base pairs (bp), with an average GC content of 38.4%. The novel *tet*(X) gene is located within a putative ICE spanning 64,344 bp, with an average GC content of 39.4% (Figure 2). The element encodes 66 ORF reading frames, encompassing components such as the type IV secretion system, type IV coupling protein, integrase, relaxase, ARGs, IS elements, and a 19 bp repeated sequence upstream and downstream, designated as ICE*EmeChn3*. Inverse PCR and Sanger sequencing confirmed that ICE*EmeChn3* can form circular intermediate, indicating its potential transferability. ICE*EmeChn3* consists of a highly conserved backbone region (43.26 kp), and a MDR region (21.06 kp) containing multiple ARGs and IS transposases. It exhibits high homology to ICE*EaIII(5)*, with 96.64% identity at 83% coverage (BK010610.1), and to the chromosome with 96.5% identity at 69% coverage (CP077751.1), in *E. anopheles* isolates. Furthermore, it exhibits 99.22% identity at 68% coverage (CP120710.1), and 98.37% identity at 82% coverage (CP094532.1), as well as 99.08% identity at 74% coverage (CP120209.1) with the chromosome of *Chryseobacterium* spp. isolates (Figure 2). These highly similar sequences, all located within the family Weeksellaceae, suggest the possibility that ICE*EmeChn3* originated from and potentially spread within this family. Despite multiple attempts in this study, the transfer of tigecycline resistance into the rifampicin-resistant recipient strains *Chryseobacterium* spp. 1–1 and 2–2 was unsuccessful.

The MDR region of ICE*EmeChn3* is 21,056 bp in length and has an average GC content of 41%, which is similar to that of the conserved region (39%). It carries multiple resistance genes, including *arr*, *floR*, *tet*(X), *aadS*, and *lnu*(B), conferring resistance to levofloxacin, florfenicol, tetracyclines, aminoglycosides, and lincomycin, respectively. The upstream of the MDR region is a 1,071 bp integrase, and the downstream is two IS*wz1* insertion sequences truncated in the same direction. It is noteworthy that within the MDR region of ICE*EmeChn3*, three sets of double-truncated IS*wz1* insertion sequences are present, flanking *floR*, *tet*(X), *aadS*, and *lnu*(B) with two sets of IS*wz1* insertion sequences in the same orientation, thus forming the structure of IS*wz1*-IS*wz1*-*orf*-*floR*-*orf*-IS*CR2*-*orf*-*tet*(X)-*orf*-*aadS*-*lnu*(B)-IS*wz1*-IS*wz1*. Downstream of *tet*(X) lies a complete IS*CR2* structure, which has been confirmed to be involved in the transposition of various *tet*(X) variants and is closely linked to the dissemination of *tet*(X). IS*wz1* is a member of the IS*91* family, which originates from *Weeksella zoohelcum*, a species belonging to the Weeksellaceae. It is worth noting that the IS*wz1* multicopy element, containing the novel *tet*(X) gene, can form two circular intermediates with sizes of 11.75 kb and 16.57 kb, respectively (Supplementary Figure S1). We selected the 16.57 kb circular intermediate for further Sanger sequencing confirmation. Furthermore, IS*wz1* and IS*wz1*-like transposases were found to occur with similarly high frequency in the MDR region of ICE*EmeChn3*-like elements in other members of the Weeksellaceae (Figure 2). They play a role in the formation of multidrug resistance islands, carrying various resistance genes, including *tet*(X2) and *tet*(X6), which may explain the formation mechanism of the Tet(X6)-Tet(X2) hybrid variant. Specifically, due to the sequence similarity between *tet*(X) genes, IS*wz1* independently mobilized *tet*(X2) and *tet*(X6), leading to the exchange of gene segments through homologous recombination, ultimately resulting in the formation of the recombinant variant.

### Phylogenetic analysis of 82 *Elizabethkingia meningoseptica* strains

**Figure 3:**
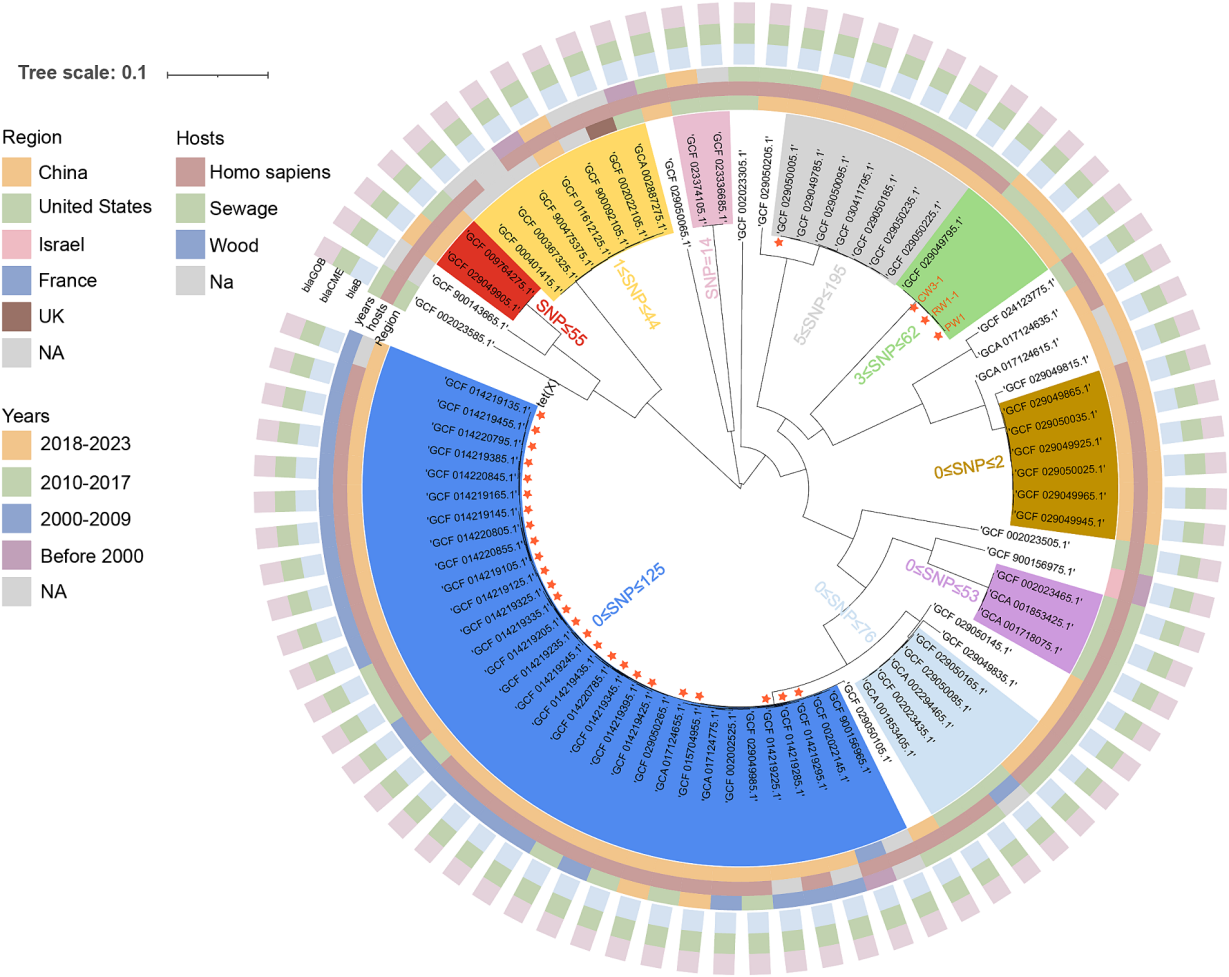
A maximum likelihood hood phylogeny based on the SNP analysis of 82 *E. meningoseptica* isolates, including 3 *tet*(X)-producing isolates collected in this study, and 79 isolates download from the NCBI genome database. Important resistance genes are represented by different shapes and colors, as illustrated in the figure. The nine clusters are shaded with different colors, and SNP differences between strains within the clusters are are indicated using Arabic numerals.

We obtained the complete genome sequences of all 79 *E. meningoseptica* strains from the NCBI genome database as of January 1, 2024, and manually excluded any duplicate submissions. Among these 79 genomes, with the exception of a few strains with missing information, the earliest traceable record dates back to 1948, and the majority of these strains originate from human or clinical sources, while only a few strains are derived from environmental origins. All 79 *E. meningoseptica* strains carried β-lactamases (*bla*_GOB_, and *bla*_CME_) and carbapenemase (*bla*_B_), explaining their intrinsic resistance to β-lactam and carbapenem (Figure 3). Additionally, 27 of the 79 strains carried *tet*(X) variants, all of which were weakly active *tet*(X2) genotype, and all originated from China. Unfortunately, the contigs containing *tet*(X2) were too short to analyze the genetic environment. Subsequently, we constructed a core genome phylogenetic tree using the *E. meningoseptica* sequence (GCF_002022145.1) as a reference, to investigate the evolutionary relationships among these 82 strains and the three strains isolated in this study. A total of 2,973,439 SNPs were used to compute the phylogenetic relationships among these isolated strains. The evolutionary tree resulted in 9 clusters (SNP < 200), each cluster containing from 2 to 25 sequences. Intriguingly, CW3-1, PW1, and RW1-1 demonstrate a clonal transmission relationship with each other, exhibiting shared resistance phenotypes and genotypes, while displaying remarkably limited SNP differences (SNP < 10) (Figure 3). We observed a geographical dissemination pattern among the *E. meningoseptica* strains, and most strains within the same clusters originate from the same country, or even the same province, which may be partly attributed to the limited number of samples of this species in the database. Importantly, CW3-1, PW1, and RW1-1 are clustered together with a *tet*(X)-negative *E. meningoseptica* (GCF_029049795.1) strain isolated from a patient in a tertiary hospital in Zhejiang Province, China in 2016, and have a close genetic relationship with each other (SNP < 62), suggesting that these three *tet*(X)-positive strains have the potential to evolve into clinically infectious strains.

## Discussion

The emergence of Tet(X) has provided the academic community with a latest model for exploring the evolution and traceability of novel ARGs. Recently classified as a distinct group from the Flavobacteriaceae family (Garcia-Lopez et al., 2019), the Weeksellaceae is regarded as the original host of *tet*(X) due to its significantly high carriage rate and similarity in GC content (Chen et al., 2020; Zhang et al., 2020). Weeksellaceae usually carries numerous FMOs with unknown functions and low homology to Tet(X) without tetracycline-degrading activity. From the perspective of genetic evolution, this aligns with the evolutionary mechanism of acquiring new functions in proteins. Before the original protein acquires a new functional phenotype, it usually needs to produce additional copies in the genome (Tawfik and Gruic-Sovulj, 2020). After the accumulation of mutations produces a new functional phenotype, the presence of additional copies will not affect the original physiological function of the original protein in the host. Weeksellaceae strains are occasionally reported to have therapeutic potential in treating diseases, but the majority of them are environmental bacteria found widely distributed in various ecological niches such as soil, sewage as well as other environments, and are potential reservoirs of multiple resistance genes. *E. meningoseptica* is a representative pathogenic bacterium in this family and can cause infections in humans and a variety of aquatic animals. Initially classified under the genus *Chryseobacterium*, molecular biology studies later led to its reclassification into a new genus, *Elizabethkingia*.

In this study, a Tet(X2) carriage rate of 27% (*n* = 22) among the *E. meningoseptica* strains highlights its significance as a reservoir. It is worth noting that different Tet(X) variants exhibit distinct host preferences, with Tet(X3) and Tet(X4) being representative variants characterized by narrow host ranges. Tet(X3) is primarily observed in *Acinetobacter* genus, while Tet(X4) predominantly exists within the Enterobacteriaceae family, specifically in *E. coli*, albeit with occasional detections in other bacterial species. Tet(X6), as a representative variant with a broad host range, exhibits significantly wider host preferences and is frequently found in coexistence with other Tet(X) variants. For instance, in our previous research, we found that Tet(X6) and Tet(X3) in *Acinetobacter* spp. are often closely linked and co-located within the IS*CR2*-mediated circularizable transposon unit (TU1) (Cui et al., 2020), forming the structure IS*CR2*-*parA*-*tet*(X3)-*tnpF*-*hp*-IS*CR2*-*hp*-Tet(X6)-*orf*-*orf*-IS*CR2*. This may explain the formation mechanism of Tet(X5) in Figure 1D, where the recombination of Tet(X6) and Tet(X3) is facilitated by IS*CR2*. Additionally, similar findings were observed in the genomes of *Bacteroidetes* and Weeksellaceae strains, where Tet(X6) and Tet(X2) coexist (Zhang et al., 2021). The close contact between them provides a plausible explanation for the occurrence of homologous recombination of the Tet(X6)-Tet(X2). The occurrence of homologous recombination among Tet(X) variants may lead to the emergence of hybrid variants with higher resistance levels or the acquisition of new resistance mechanisms, which will help increase the adaptability of *tet*(X) and expand its host range. Identifying homologous recombination events among Tet(X) variants is significant for understanding their evolutionary dynamics and dissemination potential. By recognizing instances of homologous recombination, researchers can gain deeper insights into the evolutionary relationships of Tet(X) variants and the mechanisms of exchange of homologous fragments.

The farming industry is considered an important reservoir for ARGs, which can spread within farming facilities through contact between water, soil, and animals, and ultimately pass into the food chain to affect human health. Thus far, *tet*(X)-positive strains have been documented in nearly all types of livestock and poultry farms, such as pig farms, chicken farms, waterfowl farms, and cattle farms, as well as in the surrounding environments. However, our knowledge about the prevalence of *tet*(X) within aquatic animal farming facilities, such as bullfrog farms, is limited. In this study, we visited several typical bullfrog farms in Zhejiang Province to assess their unique breeding models. To minimize expenses, bullfrog farms are often situated near natural rivers during the breeding process, as bullfrogs require a significant amount of flowing water. The breeding ponds on the farm continuously dilute and replace water by diverting upstream river water. Next, the sewage from multiple breeding ponds is collected and flows into a sedimentation tank through a sewage channel, where it undergoes initial sedimentation and is subjected to the adsorption of aquatic plants, before being finally discharged into the downstream river (Supplementary Figure S2). The commonly practiced high-density farming method in bullfrog farms, with stocking densities ranging from 15–25 kg/m^2^, inevitably contributes to the prevalence and outbreaks of infectious diseases (Di et al., 2022). To mitigate the economic losses caused by diseases, there is a significant risk of increased, and even abusive, use of antibiotics. Antibiotic use will further select and enrich multi-resistant strains, promoting the dissemination of related resistance gene clusters, plasmids, and ICEs. The multidrug resistance capacity of *E. meningoseptica* enables it to gain remarkable survival advantages in farms with complex antibiotic exposure.

Bullfrog farms are primarily characterized by a small-scale, decentralized farming model, which lacks effective wastewater treatment facilities. Zhang et al.’s study revealed that the abundance of plasmids and resistance genes significantly decreases after anaerobic digestion treatment of wastewater from large-scale pig farms, indicating the crucial role played by anaerobic digestion tanks in reducing the transmission of antibiotic resistance genes from livestock agriculture to the environment (Zhang R. M. et al., 2022). Typically, wastewater treatment systems on farms consist of a two-stage process. The primary treatment phase involves physical processes such as sedimentation tanks to remove a majority of suspended solids. The secondary treatment phase includes biological processes such as anaerobic fermentation and biofiltration to degrade complex organic compounds into non-toxic substances (Pei et al., 2019). However, the utilization of simple sedimentation methods in bullfrog farm wastewater treatment may offer limited effectiveness in mitigating the spread of ARGs into the natural environment. Previous studies have confirmed that the reduction of ARGs can be deemed negligible after the removal of suspended solids in primary treatment (Chen and Zhang, 2013). The *E. meningoseptica* clone strains isolated in this study, which were obtained from bullfrog breeding ponds, downstream discharge outlets, and downstream rivers, clearly indicate the spread from breeding facilities to the natural environment along the wastewater flow, thereby underscores the critical need for establishing wastewater treatment facilities in bullfrog farms.

In the genus *Elizabethkingia*, the majority of genomes consist of a single chromosome sequence, lacking the diverse plasmids commonly found in Enterobacteriaceae. However, the ubiquitous ICEs may have served as a main conduit assisting the exchange of genetic material between *Elizabethkingia* spp., as previous studies reported their involvement in the transfer of resistance genes through conjugation (Xu et al., 2019; Fu et al., 2021). One possible explanation for the unsuccessful transfer of tigecycline resistance in this study is the absence of suitable insertion sites in recipient strains 1–1 and 2–2. Given that Weeksellaceae are often multidrug-resistant, our selection of recipient strains is limited. This study has discovered the involvement of IS*wz1* transposon element in the recombination of ICE*EmeChn3*-like MDR regions, and its ability to assist in the dissemination of *tet*(X) within the Weeksellaceae family. This finding will enhance our understanding of the early dissemination events of *tet*(X) in ancestral hosts and provide a more comprehensive depiction of the spread pathway of *tet*(X) from environmental strains to clinically pathogenic bacteria. The emergence of the novel *tet*(X) variant in *E. meningoseptica* strains suggests that the *tet*(X) is still undergoing active evolution, underscoring the importance of continuous monitoring of *tet*(X) in various ecological niches. Future investigations, particularly in emerging farming models such as bullfrog farms, will be crucial for understanding the evolutionary drivers and transmission patterns of ARGs, as well as for providing technical support for targeted measures and policies.

## Conclusion

Overall, our study has revealed that bullfrog farms are an overlooked source of contamination, with untreated wastewater carrying antibiotic-resistant strains/genes directly released into the environment, posing potential risks. We conducted the first analysis of the prevalence of *tet*(X) in *E. meningoseptica* species (27%) and characterized a novel hybrid variant of Tet(X). Furthermore, to our knowledge, Tet(X6) serves as a cornerstone among the Tet(X) variants, and its involvement in homologous recombination between these variants contributes significantly to their rapid diversification. The findings provide valuable insights into the evolutionary dynamics of Tet(X) variants and emphasize the need for collaborative efforts to combat the evolution and dissemination of resistance genes. Moreover, this study identified the potential role of IS*wz1* in facilitating the dissemination of *tet*(X) among Weeksellaceae family, filling the gap in the spread and diffusion pathways of *tet*(X) in early hosts. In the future, close monitoring is needed to track the spread of *tet*(X) from the Weeksellaceae to other clinically relevant pathogens.

## Data availability statement

The datasets presented in this study can be found in online repositories. The names of the repository/repositories and accession number(s) can be found below: https://www.ncbi.nlm.nih.gov/, PRJNA1097367.

## Author contributions

HJ: Data curation, Writing – original draft, Formal analysis. QJ: Data curation, Formal analysis, Writing – original draft. XJ: Writing – original draft, Resources. XZ: Writing – original draft, Data curation. M-GW: Writing – original draft, Conceptualization. R-YS: Writing – original draft, Data curation. CC: Data curation, Funding acquisition, Writing – review & editing.

## Footnotes

^1^ https://cge.cbs.dtu.dk/services/ResFinder/

^2^ https://www-is.biotoul.fr

^3^ http://www.atgc-montpellier.fr/phyml/

## References

[ref1] Chan J. C. Chong C. Y. Thoon K. C. Tee N. W. S. Maiwald M. Lam J. C. M.. (2019). Invasive paediatric *Elizabethkingia meningoseptica* infections are best treated with a combination of piperacillin/tazobactam and trimethoprim/sulfamethoxazole or fluoroquinolone. J. Med. Microbiol. 68, 1167–1172. doi: 10.1099/jmm.0.001021, PMID: 31199227 PMC7423161

[ref2] Chen C. Cui C. Y. Yu J. J. He Q. Wu X. T. He Y. Z.. (2020). Genetic diversity and characteristics of high-level tigecycline resistance Tet(X) in *Acinetobacter* species. Genome Med. 12:111. doi: 10.1186/s13073-020-00807-5, PMID: 33287863 PMC7722449

[ref3] Chen H. Zhang M. (2013). Effects of advanced treatment systems on the removal of antibiotic resistance genes in wastewater treatment plants from Hangzhou, China. Environ. Sci. Technol. 47, 8157–8163. doi: 10.1021/es401091y, PMID: 23802698

[ref4] CLSI (2020). Clinical and Laboratory Standards Institute (CLSI). Performance Standards for Antimicrobial Susceptibility Testing. CLSI Supplement M100, 30th Edn. Wayne, PA: CLSI.

[ref5] Cui C. Y. Chen Q. He Q. Chen C. Zhang R. M. Feng Y.. (2022a). Transferability of tigecycline resistance: characterization of the expanding Tet(X) family. WIREs Mech Dis 14:e1538. doi: 10.1002/wsbm.1538, PMID: 35023325

[ref6] Cui C. Chen C. Liu B. He Q. Wu X. Sun R.. (2020). Co-occurrence of plasmid-mediated tigecycline and carbapenem resistance in *Acinetobacter* spp. from waterfowls and their neighboring environment. Antimicrob Agents Ch 64:e02502. doi: 10.1128/AAC.02502-19, PMID: 32122894 PMC7179582

[ref7] Cui C. He Q. Jia Q. Li C. Chen C. Wu X.. (2021). Evolutionary trajectory of the Tet(X) family: critical residue changes towards high-level tigecycline resistance. mSystems 6:50. doi: 10.1128/mSystems.00050-21, PMID: 34006624 PMC8269203

[ref8] Cui C. Y. Li X. J. Chen C. Wu X. T. He Q. Jia Q. L.. (2022b). Comprehensive analysis of plasmid-mediated *tet*(X4)-positive *Escherichia coli* isolates from clinical settings revealed a high correlation with animals and environments-derived strains. Sci. Total Environ. 806:150687. doi: 10.1016/j.scitotenv.2021.150687, PMID: 34597551

[ref9] Di Z. H. Zheng J. P. Zhang H. W. Wang X. Guo G. Y. Lin Y. Q.. (2022). Natural outbreaks and molecular characteristics of *Streptococcus agalactiae* infection in farmed American bullfrog (*Rana catesbeiana*). Aquaculture 551:737885. doi: 10.1016/j.aquaculture.2021.737885

[ref10] Fu J. Zhong C. Zhou Y. Lu M. Zong G. Zhang P.. (2021). The integrative and conjugative element ICE*CspPOL2* contributes to the outbreak of multi-antibiotic-resistant bacteria for *Chryseobacterium* spp. and *Elizabethkingia* spp. Microbiol Spectr 9:e0200521. doi: 10.1128/Spectrum.02005-21, PMID: 34937181 PMC8694125

[ref11] Garcia-Lopez M. Meier-Kolthoff J. P. Tindall B. J. Gronow S. Woyke T. Kyrpides N. C.. (2019). Analysis of 1,000 type-strain genomes improves taxonomic classification of bacteroidetes. Front. Microbiol. 10:2083. doi: 10.3389/fmicb.2019.02083, PMID: 31608019 PMC6767994

[ref12] Gasparrini A. J. Markley J. L. Kumar H. Wang B. Fang L. Irum S.. (2020). Tetracycline-inactivating enzymes from environmental, human commensal, and pathogenic bacteria cause broad-spectrum tetracycline resistance. Commun Biol 3:241. doi: 10.1038/s42003-020-0966-5, PMID: 32415166 PMC7229144

[ref13] He T. Wang R. Liu D. Walsh T. R. Zhang R. Lv Y.. (2019). Emergence of plasmid-mediated high-level tigecycline resistance genes in animals and humans. Nat. Microbiol. 4, 1450–1456. doi: 10.1038/s41564-019-0445-2 31133751

[ref14] Hu S. Chen Y. Xu H. Chen J. Hu S. Meng X.. (2023). Probability of outbreaks and cross-border dissemination of the emerging pathogen: a genomic survey of *Elizabethkingia meningoseptica*. Microbiol Spectr 11:e0160223. doi: 10.1128/spectrum.01602-23, PMID: 37815354 PMC10714787

[ref15] Hu X. Yu X. Shang Y. Xu H. Guo L. Liang Y.. (2019). Emergence and characterization of a novel IncP-6 plasmid harboring *bla*KPC-2 and *qnrS2* genes in *Aeromonas taiwanensis* isolates. Front. Microbiol. 10:2132. doi: 10.3389/fmicb.2019.02132, PMID: 31572337 PMC6751286

[ref16] Kaas R. S. Leekitcharoenphon P. Aarestrup F. M. Lund O. (2014). Solving the problem of comparing whole bacterial genomes across different sequencing platforms. PLoS One 9:e104984. doi: 10.1371/journal.pone.0104984, PMID: 25110940 PMC4128722

[ref9001] Letunic I. Bork P. (2019). Interactive Tree Of Life (iTOL) v4: recent updates and new developments. Nucleic Acids Res. 47, W256–W259. doi: 10.1093/nar/gkz239 30931475 PMC6602468

[ref17] Li H. H. Bao L. S. Deng S. M. Liu L. Cheng J. Chen X.. (2021). Investigation of *Proteus vulgaris* and *Elizabethkingia meningoseptica* invasion on muscle oxidative stress and autophagy in Chinese soft-shelled turtle (*Pelodiscus sinensis*). Sci. Rep. 11:3657. doi: 10.1038/s41598-021-83388-6, PMID: 33574492 PMC7878920

[ref18] Li R. Lu X. Peng K. Liu Z. Li Y. Liu Y.. (2020). Deciphering the structural diversity and classification of the mobile tigecycline resistance gene *tet*(X)-bearing plasmidome among bacteria. mSystems 5:e00134. doi: 10.1128/mSystems.00134-20, PMID: 32345737 PMC7190383

[ref19] Li Y. Sun X. Dong N. Wang Z. Li R. (2024). Global distribution and genomic characteristics of carbapenemase-producing *Escherichia coli* among humans, 2005-2023. Drug Resist. Updat. 72:101031. doi: 10.1016/j.drup.2023.101031, PMID: 38071860

[ref20] Lin J. N. Lai C. H. Yang C. H. Huang Y. H. (2019). *Elizabethkingia* infections in humans: from genomics to clinics. Microorganisms 7:295. doi: 10.3390/microorganisms7090295, PMID: 31466280 PMC6780780

[ref21] Lin H. Ma J. Sun J. Y. Qin Z. D. Jiang B. Li W.. (2023). Identification and characterization of *Klebsiella pneumoniae* from farmed American bullfrogs (*Rana catesbeiana*). Microbiol Spectr 11:e0357922. doi: 10.1128/spectrum.03579-22, PMID: 36602331 PMC9927386

[ref22] Liu C. Dong N. Zeng Y. Lu J. Chen J. Wang Y.. (2022). Co-transfer of last-line antibiotic resistance and virulence operons by an IncFIBk-FII-X3-ColKP3 hybrid plasmid in *Klebsiella pneumoniae*. J. Antimicrob. Chemother. 77, 1856–1861. doi: 10.1093/jac/dkac121, PMID: 35445265

[ref23] Liu M. Li X. Xie Y. Bi D. Sun J. Li J.. (2019). ICEberg 2.0: an updated database of bacterial integrative and conjugative elements. Nucleic Acids Res. 47, D660–D665. doi: 10.1093/nar/gky1123, PMID: 30407568 PMC6323972

[ref24] Liu D. Zhai W. Song H. Fu Y. Schwarz S. He T.. (2020). Identification of the novel tigecycline resistance gene *tet*(X6) and its variants in *Myroides*, *Acinetobacter* and *Proteus* of food animal origin. J. Antimicrob. Chemother. 75, 1428–1431. doi: 10.1093/jac/dkaa037, PMID: 32068864

[ref25] Ma S. Gong Y. Luo X. Peng Y. Zhang C. Zhang X.. (2023). Emerging prevalence and clinical features of *Elizabethkingia meningoseptica* infection in Southwest China: a 9-year retrospective study and systematic review. Infect Drug Resist 16, 531–543. doi: 10.2147/IDR.S397051, PMID: 36721634 PMC9884462

[ref26] Mohsin M. Hassan B. Martins W. Li R. Abdullah S. Sands K.. (2021). Emergence of plasmid-mediated tigecycline resistance *tet*(X4) gene in *Escherichia coli* isolated from poultry, food and the environment in South Asia. Sci. Total Environ. 787:147613. doi: 10.1016/j.scitotenv.2021.147613, PMID: 33992939

[ref27] Moore L. S. Owens D. S. Jepson A. Turton J. F. Ashworth S. Donaldson H.. (2016). Waterborne *Elizabethkingia meningoseptica* in adult critical care. Emerg. Infect. Dis. 22, 9–17. doi: 10.3201/eid2201.150139, PMID: 26690562 PMC4696684

[ref28] Pei M. K. Zhang B. He Y. L. Su J. Q. Gin K. Lev O.. (2019). State of the art of tertiary treatment technologies for controlling antibiotic resistance in wastewater treatment plants. Environ. Int. 131:105026. doi: 10.1016/j.envint.2019.105026, PMID: 31351383

[ref29] Robert X. Gouet P. (2014). Deciphering key features in protein structures with the new ENDscript server. Nucleic Acids Res. 42, W320–W324. doi: 10.1093/nar/gku316 24753421 PMC4086106

[ref30] Rozewicki J. Li S. Amada K. M. Standley D. M. Katoh K. (2019). MAFFT-DASH: integrated protein sequence and structural alignment. Nucleic Acids Res. 47, W5–W10. doi: 10.1093/nar/gkz342, PMID: 31062021 PMC6602451

[ref31] Sun J. Chen C. Cui C. Y. Zhang Y. Liu X. Cui Z. H.. (2019). Plasmid-encoded *tet*(X) genes that confer high-level tigecycline resistance in *Escherichia coli*. Nat Microbio 4, 1457–1464. doi: 10.1038/s41564-019-0496-4, PMID: 31235960 PMC6707864

[ref32] Sun L. Sun G. Z. Jiang Y. Mei C. Y. Wang Z. Y. Wang H. Y.. (2023). Low prevalence of mobilized resistance genes *Bla*(NDM), *mcr-1*, and *tet*(X4) in *Escherichia coli* from a hospital in China. Front. Microbiol. 14:1181940. doi: 10.3389/fmicb.2023.1181940, PMID: 37275145 PMC10237293

[ref33] Sun Q. Zhang J. Wang T. Xiong Y. Zhan X. Zhao H.. (2023). Cooking methods effectively alter perfluoroalkyl substances and nutrients in cultured and wild bullfrogs. J. Hazard. Mater. 445:130555. doi: 10.1016/j.jhazmat.2022.130555, PMID: 37055966

[ref34] Tawfik D. S. Gruic-Sovulj I. (2020). How evolution shapes enzyme selectivity – lessons from aminoacyl-tRNA synthetases and other amino acid utilizing enzymes. FEBS J. 287, 1284–1305. doi: 10.1111/febs.15199, PMID: 31891445

[ref35] EUCAST (2024). The European Committee on Antimicrobial Susceptibility Testing. Breakpoint tables for interpretation of MICs and zone diameters. Version 14.0. Available at: http://www.eucast.org

[ref36] Tsai M. A. See M. S. Chiu C. H. Wang P. C. Chen S. C. (2023). Genotypic and phenotypic analysis of *Elizabethkingia meningoseptica* in bullfrog *Rana catesbeiana* isolated in Taiwan. J. Fish Dis. 46, 1239–1248. doi: 10.1111/jfd.13842 37519120

[ref37] Wang L. Liu D. Lv Y. Cui L. Li Y. Li T.. (2019). Novel plasmid-mediated *tet*(X5) gene conferring resistance to tigecycline, eravacycline and omadacycline in clinical *Acinetobacter baumannii*. Antimicrob. Agents Chemother. 64:e01326. doi: 10.1128/AAC.01326-19, PMID: 31611352 PMC7187588

[ref38] Xu Y. Liu L. Zhang H. Feng Y. (2021). Co-production of Tet(X) and MCR-1, two resistance enzymes by a single plasmid. Environ. Microbiol. 23, 7445–7464. doi: 10.1111/1462-2920.15425, PMID: 33559156

[ref39] Xu J. Pei D. Nicholson A. Lan Y. Xia Q. (2019). In silico identification of three types of integrative and conjugative elements in *Elizabethkingia anophelis* strains isolated from around the world. mSphere 4:e00040. doi: 10.1128/mSphere.00040-19, PMID: 30944210 PMC6449604

[ref40] Zhang R. Dong N. Shen Z. Zeng Y. Lu J. Liu C.. (2020). Epidemiological and phylogenetic analysis reveals Flavobacteriaceae as potential ancestral source of tigecycline resistance gene *tet*(X). Nat. Commun. 11:4648. doi: 10.1038/s41467-020-18475-9, PMID: 32938927 PMC7494873

[ref41] Zhang R. M. Liao M. N. Wu J. E. Lu X. Q. Tan H. Z. Sun J.. (2022). Metagenomic insights into the influence of mobile genetic elements on ARGs along typical wastewater treatment system on pig farms in China. Sci. Total Environ. 839:156313. doi: 10.1016/j.scitotenv.2022.156313, PMID: 35654190

[ref42] Zhang R. M. Sun J. Sun R. Y. Wang M. G. Cui C. Y. Fang L. X.. (2021). Source tracking and global distribution of the tigecycline non-susceptible *tet*(X). Microbiol Spectr 9:e0116421. doi: 10.1128/Spectrum.01164-21, PMID: 34935428 PMC8693923

[ref43] Zhang Z. Tian X. Shi C. (2022). Global spread of MCR-producing *Salmonella enterica* isolates. Antibiotics (Basel) 11:998. doi: 10.3390/antibiotics11080998 35892388 PMC9330719

[ref44] Zhang S. Wen J. Wang Y. Wang M. Jia R. Chen S.. (2022). Dissemination and prevalence of plasmid-mediated high-level tigecycline resistance gene *tet*(X4). Front. Microbiol. 13:969769. doi: 10.3389/fmicb.2022.969769, PMID: 36246244 PMC9557194

