# Supplementary material for: Identification of novel Tet(X6)-Tet(X2) recombinant variant in Elizabethkingia meningoseptica from a bullfrog farm and downstream river in China
Source: Front Microbiol. 2024 Jul 31;15:1453801. doi: 10.3389/fmicb.2024.1453801 (PMC11322121; doi:10.3389/fmicb.2024.1453801)
Supplement: Supplementary file 1 [file Data_Sheet_1.pdf]

Supplementary Information

Table S1. MICs of 5 tetracyclines for the studied strains.

Strains	MIC (mg/L)				
	TET	DOX	MIN	TGC	ERA
E. coli TOP10+pBAD24	0.5	0.5	0.25	0.03	0.008
E. coli TOP10+pBAD24-*tet*(X2)	32	8	1	0.25	0.125
E. coli TOP10+pBAD24-*tet*(X3)	128	32	16	8	4
E. coli TOP10+pBAD24-*tet*(X4)	128	32	16	8	4
E. coli TOP10+pBAD24-*tet*(X)-novel	32	16	4	4	2

TET, tetracycline; DOX, doxycycline; MIN, minocycline; TGC, tigecycline; ERA, Eravacycline.

Table S2 Inverse primers used in this study.

Primer	Nucleotide sequence (5' to 3')	Size (bp)	Reference
ICE*EmeChn3*-F	ACCTTATGAAGGGCAAATCGGA	919	This study
ICE*EmeChn3*-R	CCAAGGAAGAGGGCGACAAT		
M1-F	GGTTCTGGAACTTGCTACTGGA	2901	This study
M2-F	CGACAGGAATTTGCTCACGC	3132	This study
M1/M2-R	TTGATACGGTTGCACCTGCT		

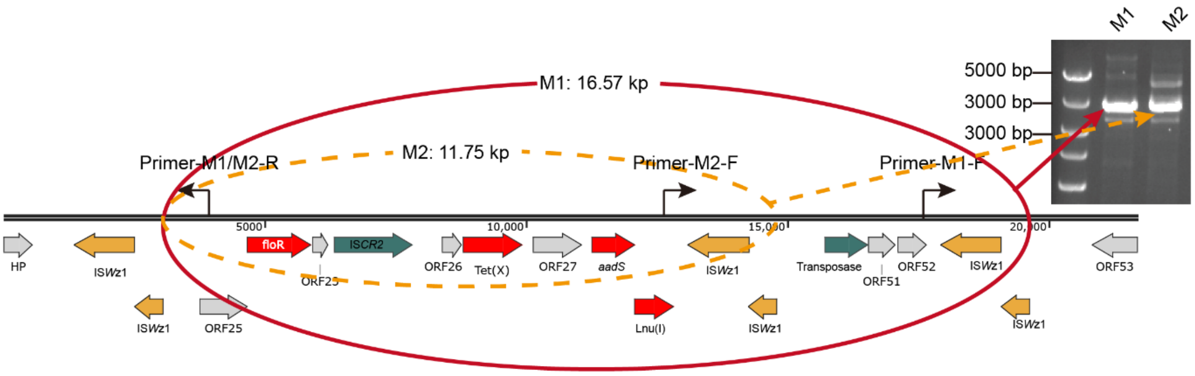

Figure S1 Schematic diagram of inverse PCR identification of two circular intermediates (M1 and M2) mediated by IS*wz1*; their structures and primer design positions are shown in the figure.

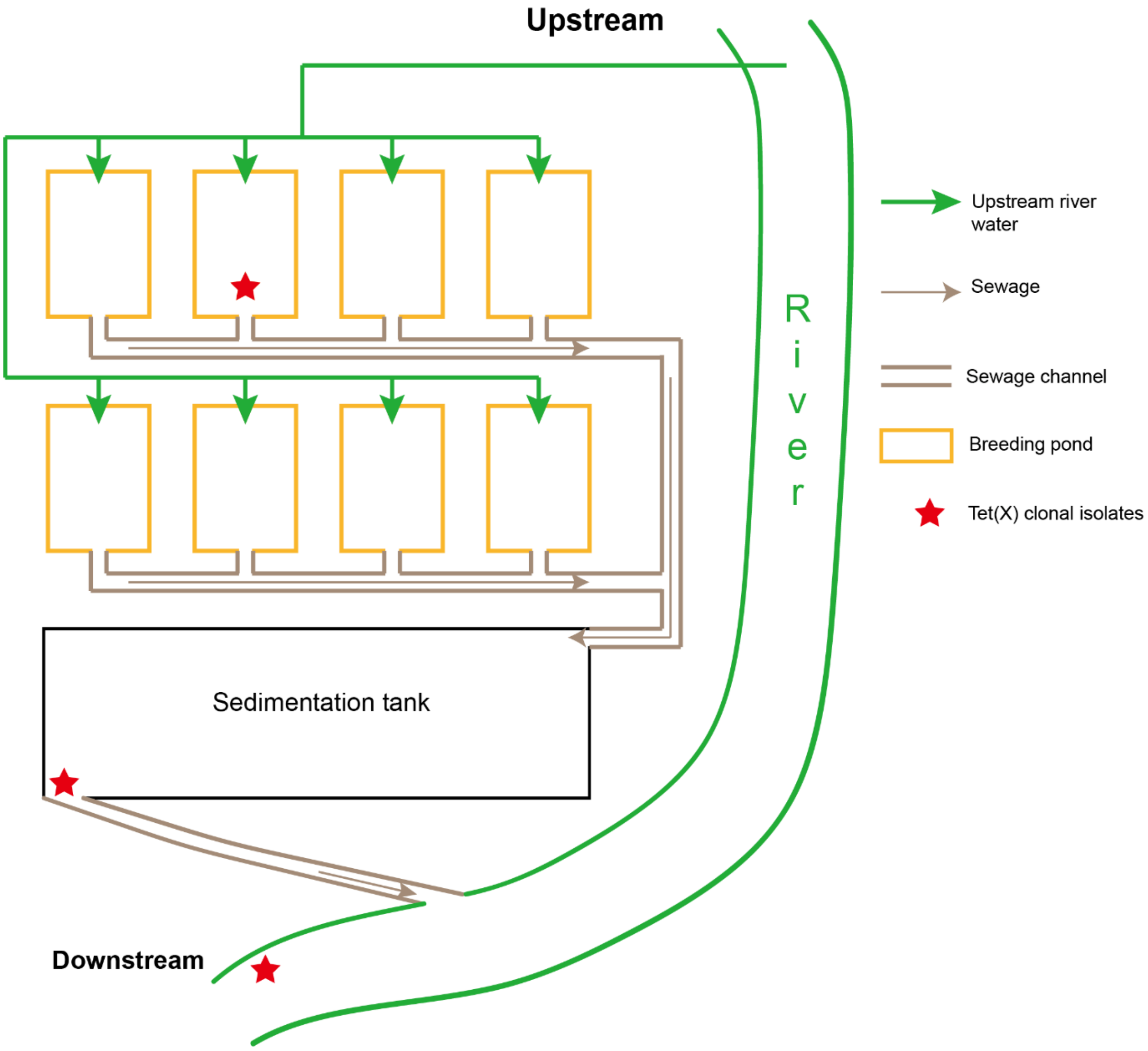

Figure S2 The diagram illustrates the drainage system of a bullfrog farm's aquaculture wastewater. The positions where three *tet*(X)-positive strains were isolated are marked with red stars.
